# Links between *S*‐adenosylmethionine and Agr‐based quorum sensing for biofilm development in *Listeria monocytogenes* EGD‐e

**DOI:** 10.1002/mbo3.1015

**Published:** 2020-03-05

**Authors:** Yue‐Jia Lee, Chinling Wang

**Affiliations:** ^1^ Department of Basic Sciences College of Veterinary Medicine Mississippi State University Mississippi State MS USA

**Keywords:** activated methyl cycle, biofilm, extracellular polymeric substances, peptidoglycan synthesis, quorum sensing

## Abstract

*Listeria monocytogene*s is the causative agent of human listeriosis which has high hospitalization and mortality rates for individuals with weakened immune systems. The survival and dissemination of *L. monocytogenes* in adverse environments can be reinforced by the formation of biofilms. Therefore, this study aimed to understand the mechanisms underlying listerial biofilm development. Given that both nutrient availability and quorum sensing (QS) have been known as the factors influencing biofilm development, we hypothesized that the signal from a sentinel metabolite *S*‐adenosylmethionine (SAM) and Agr‐based QS could be synchronous in *L. monocytogenes* to modulate nutrient availability, the synthesis of extracellular polymeric substances (EPSs), and biofilm formation. We performed biofilm assays and quantitative real‐time PCR to investigate how biofilm volumes and the expression of genes for the synthesis of EPS were affected by SAM supplementation, *agr* deletion, or both. We found that exogenously applied SAM induced biofilm formation and that the expression of genes encoding the EPS synthesis machineries was regulated by SAM and/or Agr QS. Moreover, the gene transcription of components acting in the methyl cycle for SAM synthesis and Agr QS was affected by the signals from the other system. In summary, we reveal an interconnection at the transcriptional level between metabolism and QS in *L. monocytogenes* and highlight the critical role of metabolite‐oriented QS in biofilm development.

## Graphical abstract

This study revealed mechanisms underlying EPS synthesis for biofilm development of *Listeria monocytogenes.* Especially, it elaborated on the cooperation of signal molecules in metabolic pathways and the quorum sensing (QS) network. These findings pave a way for systematically targeting key regulators in *L. monocytogenes* metabolism and QS to prevent biofilm development.

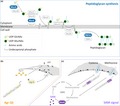

## 1 INTRODUCTION

As an environmental pathogen, *Listeria monocytogenes* replicates and survives in both the environment and within mammalian hosts (Xayarath & Freitag, 2012). Its widespread distribution makes this foodborne pathogen difficult to control and a threat to public health. Such pathogens can survive in the environment by forming surface‐associated communities called biofilms (Gutiérrez et al., 2012; Korber, Choi, Wolfaardt, Ingham, & Caldwell, 1997; Poimenidou et al., 2009). Within biofilms, the bacteria are enclosed in self‐produced extracellular polymeric substances (EPSs), enabling them to sense and adapt to diverse environments (Hall‐Stoodley, Costerton, & Stoodley, 2004).

**Figure 1:**
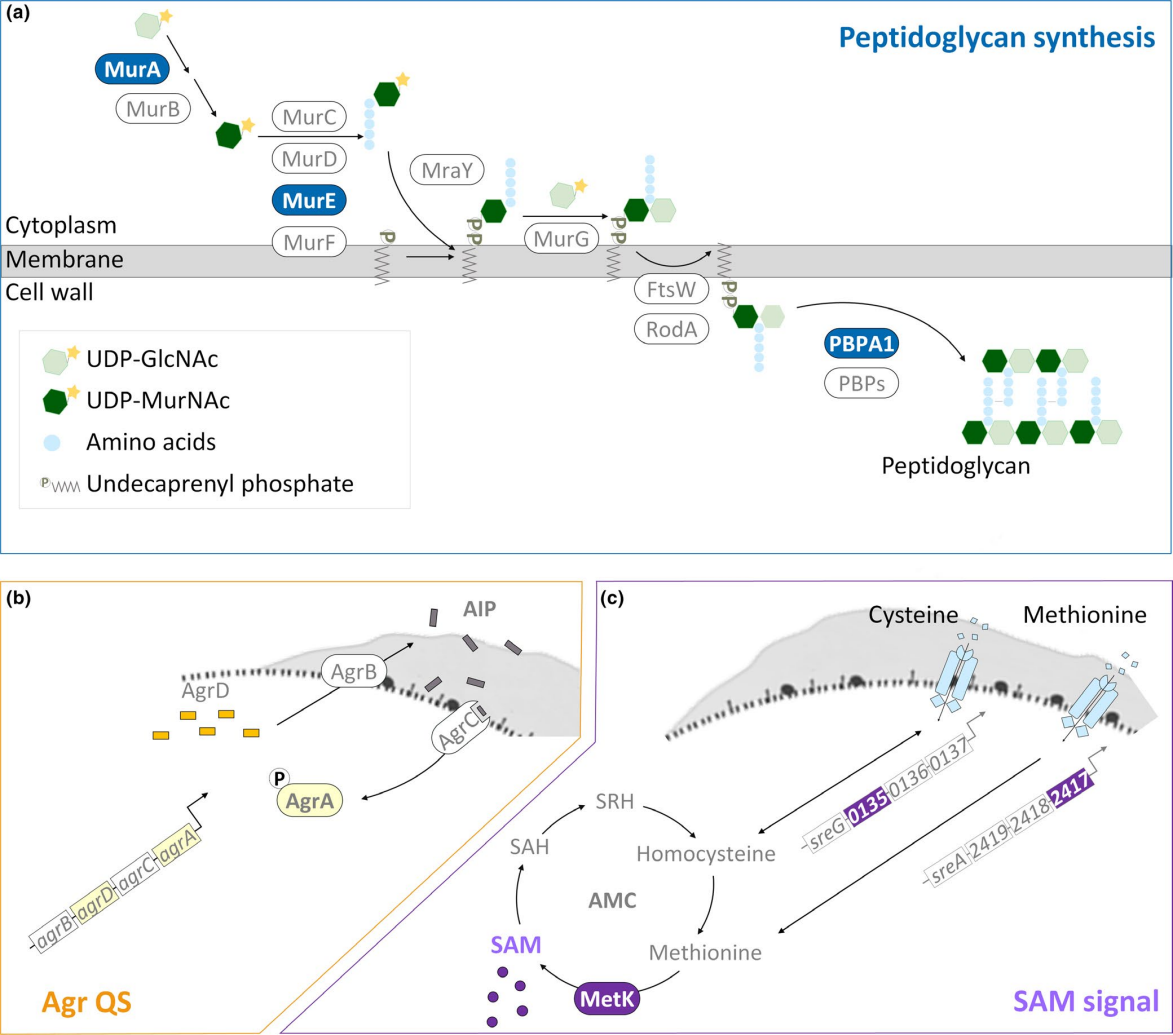
Schematic representation of the machinery of peptidoglycan synthesis, Agr quorum sensing (QS), and SAM synthesis in *Listeria monocytogenes*. Peptidoglycan synthesis is one of the main mechanisms for *Listeria* EPS synthesis. The peptidoglycans compose parts of the cell wall glycopolymers. (a) Peptidoglycan synthesis includes three stages: assembly, translocation, and polymerization of glycan units. *mur* genes and *pbp* genes are those encoding enzymes for the assembly (initial stage) of glycan monomers (from UDP‐GlcNAc to UDP‐MurNAc‐pentapeptide) and polymerization (final stage) of peptidoglycans, respectively. (b) In the accessory gene regulator (*agr*) locus‐encoding QS, AgrD is processed by AgrB to form the signal molecule AIP, and AIPs activate the two‐component system AgrCA for downstream gene regulation. (c) *S*‐adenosylmethionine (SAM) is derived from methionine by enzyme MetK in the activated methyl cycle (AMC). Two amino acid transporters import cysteine and methionine for the resources of the AMC. *lmo0135* and *lmo2417* are two genes encoding the substrate‐binding unit of transporters for cysteine and methionine, respectively

Polysaccharides and proteins are predominant molecules of EPS, together with other minor components, representing the three‐dimensional scaffold of the biofilm for mechanical stability of biofilms and the adhesion of bacterial cells to surfaces (Flemming & Wingender, 2010). Because of that composition of EPS, the production of EPS is closely linked to the synthesis of polysaccharides and peptidoglycans (polysaccharides linked with peptide bridges). Both Gram‐positive and Gram‐negative bacteria conserve a three‐stage mechanism of peptidoglycan synthesis. This process (Figure 1a) begins in the cytoplasm with the conversion of saccharide units (from UDP‐*N*‐acetylglucosamine [UDP‐GlcNAc] to UDP‐*N*‐acetylmuramic acid [UDP‐MurNAc]) and the addition of peptide bridges by proteins encoded by *mur* genes (*murA‐F*). The second step is the assembly and translocation of the lipid II precursor. MraY and MurG transfer UDP‐MurNAc‐pentapeptide and UDP‐GlcNAc to the undecaprenyl phosphate (lipid carrier) to generate lipid II. Sequentially, lipid II is translocated across the membrane through FtsW/RodA, proteins of SDES family. The process ends with the polymerization of peptidoglycan by penicillin‐binding proteins (PBPs) at least including PBPA1 (Typas, Banzhaf, Gross, & Vollmer, 2011; van Heijenoort, 2001). Previous works about mutations in the encoding genes for peptidoglycan synthesis (Wen, Bitoun, & Liao, 2015; Yong, Jing, Yuqing, Blakely, & Min, 2012) and disturbance of peptidoglycan assembly with small molecules (Kolodkin‐Gal et al., 2010; Parsons, Costolo, Brown, & Kathariou, 2017) confirmed the essential role of peptidoglycan synthesis in biofilm development.

Transcriptomic studies recently verified that biofilms comprise heterogeneous populations of bacteria with differences in replication rates and gene regulation between the sessile and planktonic cells (Hamilton et al., 2009; Lazazzera, 2005; Luo et al., 2013). This suggests that the bacterial population takes the advantage of the heterogeneous nature of the biofilm to survive under environmental stresses. For example, bacteria within biofilms which are in sessile life mode regulate the expression of genes for higher tolerance to antimicrobial treatments (Chavant, Gaillard‐Martinie, & Hébraud, 2004; Davies, 2003; Folsom et al., 2010). For *L. monocytogenes*, such coordination of gene expression for biofilm development (Garmyn, Augagneur, Gal, Vivant, & Piveteau, 2012; Lauderdale, Boles, Cheung, & Horswill, 2009) has been attributed to quorum sensing (QS), a cell‐to‐cell communication system for the synthesis, secretion, and detection of small signal molecules. One of the QS systems in *L. monocytogenes* is encoded by the accessory gene regulator (*agr*) locus—*agrBDCA* (Figure 1b). Four proteins compose the Agr‐based QS system (Agr QS). The membrane protein AgrB turns the signal precursor AgrD into autoinducing peptide (AIP) and translocates AIP outside the cell. AIP is recognized by the histidine kinase AgrC of the classical two‐component system (AgrCA), and the signal is transduced by the transcriptional regulator AgrA to the downstream genes including those for biofilm formation (Rieu, Weidmann, Garmyn, Piveteau, & Guzzo, 2007) and virulence (Autret, Raynaud, Dubail, Berche, & Charbit, 2003; Riedel et al., 2009). Although the transcriptional regulation of Agr QS on virulence genes has been studied extensively (Garmyn et al., 2012; Pinheiro et al., 2018; Riedel et al., 2009), how the genes for peptidoglycan synthesis, a part of the resources for EPS, are regulated by Agr QS is less clear in *L. monocytogenes*.

Nutrient availability also strongly influences biofilm development of *L. monocytogenes* (Helloin, Jänsch, & Phan‐Thanh, 2003; Zhou et al., 2012). As an intermediate metabolite in the activated methyl cycle (AMC), *S*‐adenosylmethionine (SAM) generated from methionine via the synthase MetK is recognized as a sentinel metabolite (Figure 1c). SAM is not only a methyl donor for the methylation of macromolecules (Parveen & Cornell, 2011) but also an effector molecule for riboswitches which are certain 5' UTRs controlling the expression of their downstream genes based on the binding with SAM (Breaker, 2012). Genes encoding *S*‐adenosylmethionine synthetase (*metK*) and the substrate‐binding subunit of the transporter of methionine (*lmo2417*) and cysteine (*lmo0135*) are parts of those downstream metabolic genes regulated by SAM riboswitches and required for the balance of AMC (Loh et al., 2009; Toledo‐Arana et al., 2009; Winkler, Nahvi, Sudarsan, Barrick, & Breaker, 2003). Because of the properties of SAM, variations in SAM levels could affect a variety of cellular functions and the regulation of SAM signal could be used to harmonize these various functions.

To advance our understanding of the mechanisms underlying *L. monocytogenes* biofilm formation, we investigated the role of SAM signal in this process by supplementing SAM during biofilm formation. Since previously published studies have linked Agr QS to metabolic pathways (Pinheiro et al., 2018; Pohl et al., 2009), we further tested the hypothesis that the SAM signal may interact with Agr QS to cooperatively regulate *L. monocytogenes* biofilm formation. Here, we showed that SAM supplement induced biofilm formation under nutrient limitation, revealing a metabolic role of the AMC for *L. monocytogenes* biofilm formation. Notably, we identified the peptidoglycan synthesis‐associated genes regulated by the SAM signal and/or Agr QS. We also found that the SAM signal and Agr QS were mutually regulated at the transcriptional level. These suggest redundant regulations by the SAM signal and Agr QS on the synthesis of EPS in *L. monocytogenes*. Furthermore, our results indicated that the manipulated objects in this mutual regulation were dependent on the transition from the planktonic to sessile life mode.

## 2 MATERIALS AND METHODS

### 2.1 Bacterial strain and culture conditions

*Listeria monocytogenes* strain EGD‐e (serovar 1/2a) was used in this study, as serovar 1/2a strains account for >50% of the *L. monocytogenes* isolates recovered from foods and the environment (Aarnisalo et al., 2003; Gilbreth et al., 2005). The mutants with in‐frame deletions of *agrA* (Δ*agrA*) and *agrD* (Δ*agrA*) were derived from EGD‐e and kindly provided by Dr. Pascal Piveteau (Rieu et al., 2007). For all assays, the bacteria were precultured in brain heart infusion (BHI) broth (Difco) agitatedly for 16 hr at 37°C.

### 2.2 Biofilm formation in the presence or absence of *S*‐adenosylmethionine (SAM)

*Listeria monocytogenes* (wild type, Δ*agrA*, and Δ*agrD*) cells were centrifuged, and the pellets were diluted to 10^7^ CFU/ml based on plate enumeration. A 200‐μl aliquot of each strain was inoculated into 96‐well polystyrene microtiter plates (CELLTREAT) with BHI broth or 10% BHI broth containing 250 and 500 μM membrane‐permeable *S*‐(5'‐adenosyl)‐l‐methionine p‐toluenesulfonate salt (SAM; Sigma). For RNA extraction from biofilm cultures, a 5‐ml aliquot of each strain was inoculated in 6‐well polystyrene microtiter plates. The plates were incubated statically at 37°C for 24 hr.

### 2.3 Quantitative assay for biofilm formation

The biofilms formed on the surfaces of wells were measured using crystal violet staining as previously described (Lourenço, Rego, Brito, & Frank, 2012) with minor modifications. Briefly, after the suspension was removed, the wells were air‐dried and stained with 200 μl of 0.1% crystal violet solution including 20% ethanol for 30 min at room temperature. Unbound dye was removed by rinsing three times with 200 μl sterile double‐distilled water, followed by a 30‐min air dry. Crystal violet bound to biofilms was solubilized in 200 μl 10% acetic acid with 100 rpm agitation. OD_595_ was measured using a Synergy HT microplate reader (BioTek).

### 2.4 Preparation of planktonic cells

*Listeria monocytogenes* (wild type, Δ*agrA*, and Δ*agrD*) cells were centrifuged, and the pellets were diluted to 10^7^ CFU/ml with BHI broth based on plate enumeration. A 5‐ml aliquot of each strain was inoculated into 50‐ml conical centrifuge tubes. The tubes were incubated under the agitated condition (200 rpm) at 37°C for 24 hr.

### 2.5 RNA extraction and reverse transcription‐quantitative PCR (RT‐qPCR)

The pellets of sessile cells from biofilm cultures and planktonic cells growing under the agitated condition were resuspended in lysis buffer (15 mg/ml lysozyme and 200 µg/ml proteinase K in TE buffer) and incubated at 37°C for 10 min. The resultant samples were transferred to a lysing matrix B tube (MP Biomedicals) and vortexed for 15 s for four times using a disruptor (Scientific Industries) with a 1‐min pause on ice between vortexes. Total RNA was extracted from the cells using acid phenol–chloroform extraction (Chomczynski & Sacchi, 2006). Five units of RNase‐free DNase (Promega) was applied to the samples at 37°C for 15 min before purification with an RNeasy Plus Universal Mini Kit (Qiagen). The purity and concentration of RNA were determined by gel electrophoresis and a NanoDrop ND‐1000 UV‐Visible Light Spectrophotometer. One‐microgram aliquots of RNA samples were reverse‐transcribed to cDNA using a SuperScript VILO cDNA Synthesis Kit (Qiagen). cDNA diluted by a factor of 5, 10, or 20 was used as the template in a 10 μl reaction mixture containing the primers listed in Table 1. qPCR was performed with a SYBR Green Master Kit (Applied Biosystems) under the following conditions: 95°C for 2 min, followed by 40 cycles of 95°C for 5 s and 60°C for 30 s on a 7,500 Fast Real‐Time PCR System (Applied Biosystems). *L. monocytogenes* 16S rRNA was used as an internal control. The relative changes in mRNA expression were analyzed by the 2^−∆∆^ *^CT^* method.

**Table 1:** Target genes and their aligned primers used in this study

Relative pathway	Name of locus	Locus tag	Protein function	Primer
Agr QS	*agrA*	lmo0051	Response regulator of a two‐component system	F: GAAGATAACAGAATGCAGCGAGAAAGG R: GGATCAAACTTCCGAATTTCCTGAGC
	*agrB*	lmo0048	Protease performing the proteolytic processing of quorum sensing signal molecule precursor	F: GCTTATTGATGTTTGTGCTTGCGC R: GTGTTCTTCACCGATTAAAGGCAAAC
	*agrC*	lmo0050	Histidine kinase of a two‐component system	F: GTAGTTTCAGCTTCTATTACGCTTGTG R: ATACCAACAAATTCGCCAACATTCC
	*agrD*	lmo0049	Quorum sensing signal molecule precursor	F: GAATAAATCAGTTGGTAAATTCCTTTCTAG R: CAAATGGACTTTTTGGTTCGTATACAAAC
Synthesis of SAM signal	—	lmo2417	ABC transporter substrate‐binding protein for methionine transport system	F: ATGCTGGAAGTAGTTAGCGTCTAAG R: ATCCAATACACCACATGCCCAAATC
	—	lmo0135	ABC transporter substrate‐binding protein for cysteine transport system	F: GCAGACTACTCTATCGCACTAAATGG R: GATTTCTTGACGTTCTTTGTCGTCAGC
	*metK*	lmo1664	*S*‐adenosylmethionine synthetase	F: TCACTTCTGGGAAAAGATACGTGTG R: CGCATGGTTTAGCTCGCAAATTAAC
Peptidoglycan synthesis	*murA*	lmo2526	UDP‐N‐acetylglucosamine 1‐carboxyvinyltransferase for the addition of enolpyruvyl to UDP‐N‐acetylglucosamine	F: AAGTTACAAGGAGCAGAAGTTGCAG R: TACATCGACTTTGGAATCATCTACACG
	*murE*	lmo2038	UDP‐N‐acetylmuramoylalanyl‐D‐glutamate‐‐2,6‐diaminopimelate ligase for the addition of meso‐diaminopimelic acid to UDP‐N‐acetylmuramoyl‐l‐alanyl‐d‐glutamate	F: TGTTTCTTGTAAAGTTAGGCTGTCTGG R: CGTTAAAACTCGTTGGGATTACTGGG
	*pbpA1*	lmo1892	Class A penicillin‐binding protein (A1) catalyzing transglycosylation and transpeptidation of peptidoglycans	F: AGAGTACACGGAGAAAATGCTCAATAC R: TGGTTTCATAGTAGACCCAACAGAAC
—	*16s rRNA*	lmor01	Small subunit of ribosome	F: GAGGGTCATTGGAAACTGGAAGAC R: CCTAACACTTAGCACTCATCGTTTACG

### 2.6 Statistical analysis

Each experiment was repeated at least three times. The significance of the differences among groups was assessed by one‐way analysis of variance (ANOVA) using SigmaPlot (Systat Software). Pairwise comparisons were performed by using Tukey's test, and the differences were marked by lowercase letters. Student's *t* test was applied to determine a significant difference (marked by *) between two sets of data. For all tests, a *p* value of <.05 was considered significant.

## 3 RESULTS

### 3.1 SAM enhanced *L. monocytogenes* biofilm formation

**Figure 2:**
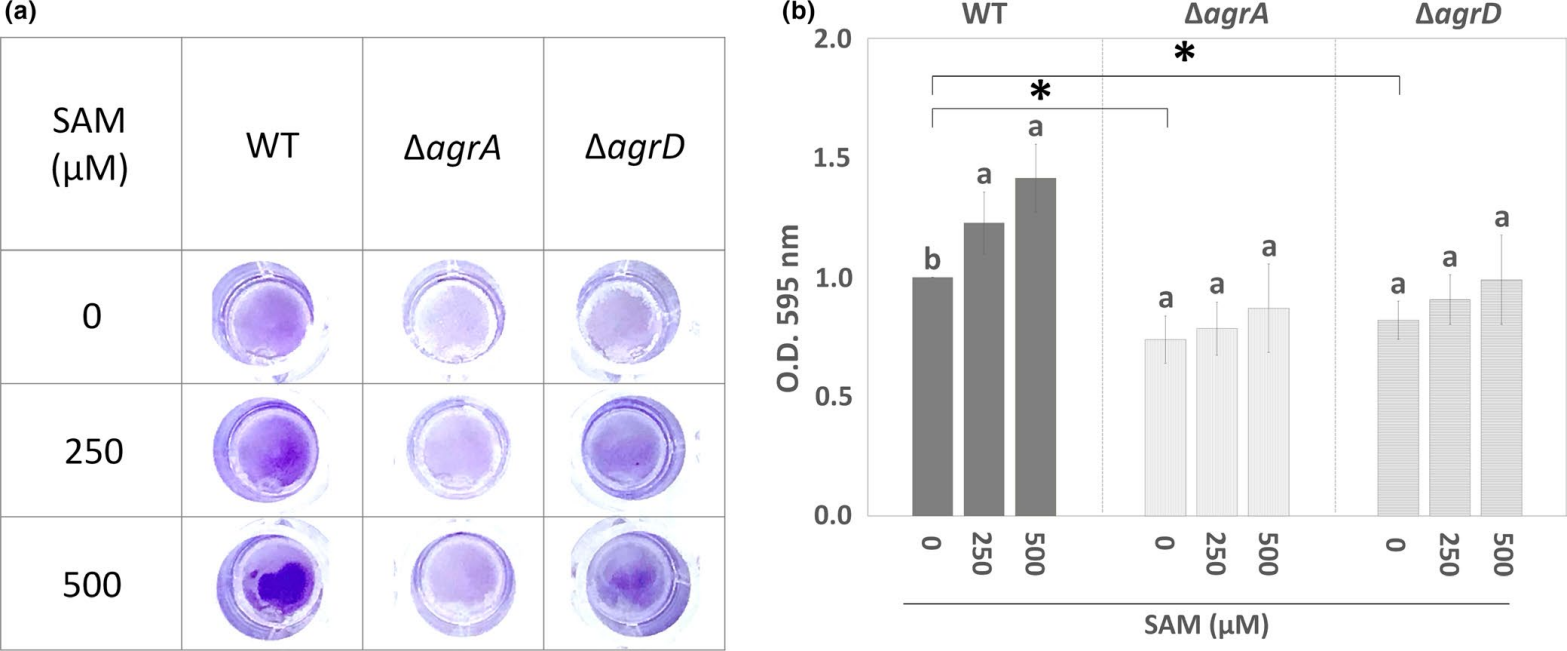
Visualization and quantification of *Listeria monocytogenes* biofilm formation in the presence or absence of SAM and the deficient Agr system. The wild‐type (WT) strain and two mutants with in‐frame deletion of *agrA* and *agrD* (Δ*agrA* and Δ*agrD*) were incubated in the presence or absence of SAM under the static condition to form biofilms. (a) Biofilm biomass was stained with crystal violet solution. (b) The stained biofilm biomass was quantified based on the optical density at 595 nm. Data are means ± standard errors from three independent experiments with three replicates for each experiment. For three groups treated with 0, 250, and 500 µM SAM within a single strain (WT, *ΔagrA,* or *ΔagrD*), the same lowercase letter above any two groups indicates that the difference between their means is not statistically significant. Asterisks (*) indicate significant differences between the two groups pointed out by brackets (*p* < .05)

To test the hypothesis that changes in SAM level can affect *L. monocytogenes* biofilm formation, we measured biofilm biomass formed by the wild‐type (WT) strain and the mutants with in‐frame deletion of *agrA* (Δ*agrA*) and *agrD* (Δ*agrD*) in the presence or absence of SAM with crystal violet staining method. The biofilm biomass of WT cultured under nutrient limitation (10‐fold diluted BHI) was dose‐dependently increased with the addition of SAM (Figure 2a). The quantified data showed that *L. monocytogenes* biofilm biomass was increased around 1.5‐fold in the presence of 500 µM SAM (Figure 2b). Compared to WT, the biofilm biomass of Δ*agrA* and Δ*agrD* mutants was significantly reduced. Moreover, SAM treatment was unable to significantly enhance biofilm biomass of the Δ*agrA* and Δ*agrD* mutants. This indicated that the deficiency in the Agr QS system compromised SAM‐enhanced biofilm formation, suggesting a link between intracellular SAM signal and Agr QS.

### 3.2 SAM upregulated the expression of genes for Agr QS and peptidoglycan synthesis

**Figure 3:**
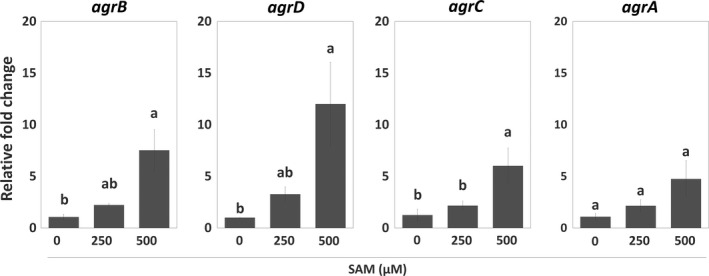
Regulation of genes associated with Agr QS during *Listeria monocytogenes* biofilm formation in the presence or absence of SAM. The wild‐type (WT) strain was incubated in the presence or absence of SAM under the static condition to form biofilms. Total RNA was extracted from sessile WT cells for gene expression analysis using qPCR. Relative changes in the expression of *agr* locus (*agrBDCA*) were calculated by setting the value from the group of WT without SAM treatment (0 µM SAM) as 1. Data are means ± standard errors from at least three independent experiments with three replicates for each experiment. For three groups treated with 0, 250, and 500 µM SAM within a single gene (*agrB*, *agrD*, *agrC,* or *agrA*), the same lowercase letter above any two groups indicates that the difference between their means is not statistically significant (*p* < .05)

**Figure 4:**
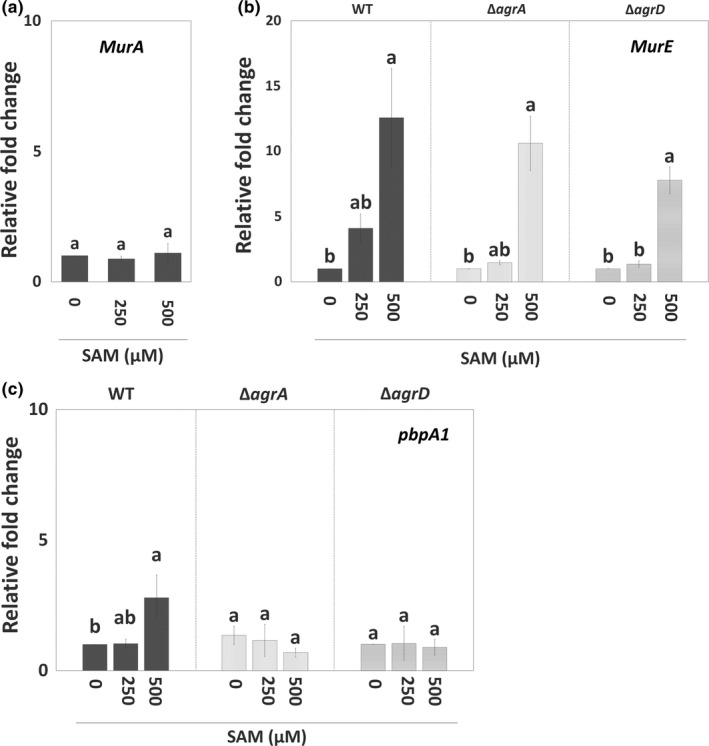
Genes associated with peptidoglycan synthesis were regulated by SAM signal and the Agr QS. The wild‐type (WT) strain as well as Δ*agrA* and Δ*agrD* mutants were incubated in the presence or absence of SAM under the static condition to form biofilms. Total RNA was extracted from sessile cells of WT, Δ*agrA,* and Δ*agrD* for gene expression analysis using qPCR. Relative changes in the expression of *murA* (a), *murE* (b), and *pbpA1* (c) for canonical peptidoglycan synthesis were calculated by setting the value from the group of WT, Δ*agrA,* or Δ*agrD* without SAM treatment (0 µM SAM) as 1. Data are means ± standard errors from at least three independent experiments with three replicates for each experiment. For three groups treated with 0, 250, and 500 µM SAM within a single gene (*murA*, *murE,* or *pbpA1*), the same lowercase letter above any two groups indicates that the difference between their means is not statistically significant (*p* < .05)

To further understand how the SAM signal interacts with Agr QS and regulates EPS synthesis during biofilm formation, we analyzed the expression of *agr* genes and genes encoding components for peptidoglycan synthesis in sessile WT with or without SAM treatment. In the presence of SAM, *agr* locus was significantly induced. Of this locus, *agrD* expression was upregulated the most, while *agrA* expression was slightly increased (Figure 3). Regarding peptidoglycan synthesis, the expression of *murA* was not affected by the treatment of SAM (Figure 4a). By contrast, *murE* and *pbpA1*, which are responsible for the assembly (initial stage) and polymerization (final stage) of peptidoglycans, had their expression increased in the sessile WT cells as the concentration of supplemental SAM increased at the onset of biofilm formation (Figure 4b,c). We further tested the regulation of Agr QS on SAM‐dependent expression of *murE* and *pbpA1* in *agr* mutants treated with SAM. In sessile Δ*agrA* and Δ*agrD* cells, the treatment of SAM similarly increased *murE* expression (Figure 4b) but not *pbpA1* expression (Figure 4c). In other words, SAM‐induced *murE* transcription was independent with Agr QS, while *pbpA1* transcription could be regulated by both SAM signal and Agr QS.

### 3.3 Agr QS affected the expression of genes for the synthesis of peptidoglycan and SAM signal

**Figure 5:**
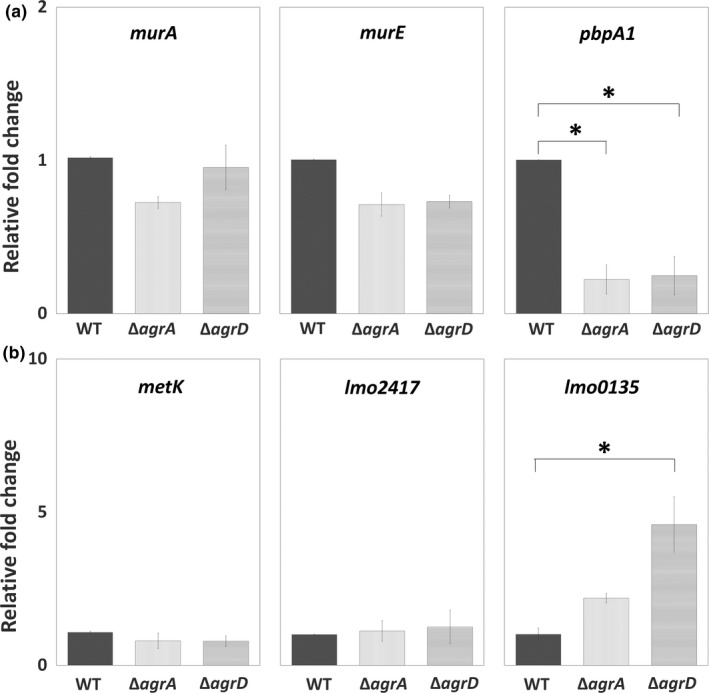
Expression of genes associated with the synthesis of peptidoglycan and SAM signal in sessile WT*,* Δ*agrA,* and Δ*agrD* cells. The WT as well as Δ*agrA* and Δ*agrD* mutants were incubated under the static condition to form biofilms. Total RNA was extracted from sessile cells of WT, Δ*agrA,* and Δ*agrD* for gene expression analysis using qPCR. Relative changes in the expression of *murE* and *pbpA1* for peptidoglycan synthesis (a) and *metK*, *lmo2417* and *lmo0135* for synthesizing SAM and importing methionine or cysteine (b) were calculated by setting the value from the group of sessile WT cells as 1. Data are means ± standard errors from at least three independent experiments with three replicates for each experiment. An asterisk (*) indicates the significant difference between the two groups pointed out by a bracket (*p* < .05)

To investigate the transcriptional regulation of Agr QS on the synthesis of peptidoglycan (*murA*, *murE*, and *pbpA1*) and SAM signal (*metK*, *lmo2417*, and *lmo0135*), we tested and compared the expression of target genes for these pathways among WT and two mutants, Δ*agrA* and Δ*agrD*. Of three tested genes for peptidoglycan synthesis, the expression of *pbpA1* was significantly repressed in sessile Δ*agrA* and Δ*agrD* cells compared with sessile WT cells, while the expression of *murA* and *murE* stayed at similar levels among WT and two mutants (Figure 5a). For the synthesis of SAM signal, the expression of *metK* and *lmo2417*, responsible for synthesizing SAM and importing methionine, was not noticeably altered by the lack of Agr QS. However, the expression of *lmo0135*, responsible for importing cysteine, was induced by the lack of Agr QS, although a significant induction was shown in sessile Δ*agrD* cells only (Figure 5b).

### 3.4 The regulation of Agr QS was dependent with bacterial life modes

**Figure 6:**
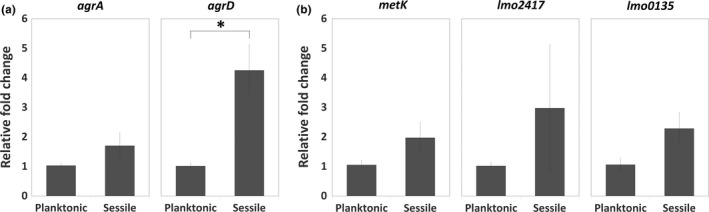
Expression of genes associated with Agr QS and the synthesis of SAM signal in planktonic or sessile WT cells. WT was incubated under the agitated and static condition for the collection of planktonic and sessile cells, respectively. Total RNA was extracted from planktonic and sessile WT cells for gene expression analysis using qPCR. Relative changes in the expression of *agrA* and *agrD* in Agr QS (a) and *metK*, *lmo2417*, and *lmo0135* for the synthesis of SAM signal (b) were calculated by setting the value from the group of planktonic WT cells as 1. Data are means ± standard errors from at least three independent experiments with three replicates for each experiment. An asterisk (*) indicates the significant difference between the two groups pointed out by a bracket (*p* < .05)

**Figure 7:**
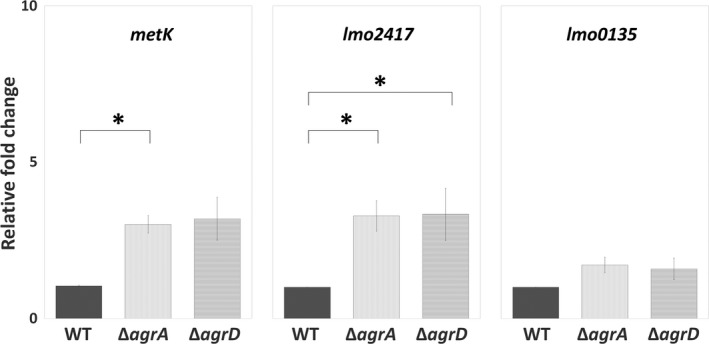
Expression of genes associated with the synthesis of SAM signal in planktonic WT*,* Δ*agrA,* and Δ*agrD* cells. The WT as well as Δ*agrA* and Δ*agrD* mutants were incubated under the agitated condition to keep in the planktonic life mode. Total RNA was extracted from planktonic cells of WT, Δ*agrA,* and Δ*agrD* for gene expression analysis using qPCR. Relative changes in the expression of *metK*, *lmo2417,* and *lmo0135* for synthesizing SAM and importing methionine or cysteine were calculated by setting the value from the group of planktonic WT cells as 1. Data are means ± standard errors from at least three independent experiments with three replicates for each experiment. An asterisk (*) indicates the significant difference between the two groups pointed out by a bracket (*p* < .05)

Considered that bacterial physiology undergoes a dramatic change during biofilm formation, we prompted to assess the effect of bacterial life mode on Agr QS and the SAM signal. The expression levels of the first and last gene in the *agr* locus (*agrA* and *agrD*) as well as genes involved in the cycle of SAM production (*metK*, *lmo2417*, and *lmo0135*) were compared between the planktonic and sessile life modes. We found that the expression of *agrD* was significantly higher in sessile WT cells than in planktonic WT cells, while the expression of *agrA* as well as SAM production‐related genes *metK*, *lmo2417*, and *lmo0135* was similar in both sessile and planktonic WT cells (Figure 6). Given that the switch of bacterial life mode affected the level of *agrD*, we hypothesized that Agr QS transcriptional regulation on the genes (*metK*, *lmo2417*, and *lmo0135*) for the SAM production, *that is,* the link between Agr QS and SAM signal, would be different based on bacterial life modes. It is interesting that the expression of *metK* and *lmo2417*, instead of *lmo0135* which was induced in sessile mutants (Figure 5b), was upregulated in planktonic Δ*agrA* and Δ*agrD* cells compared with planktonic WT cells (Figure 7).

## 4 DISCUSSION

The persistence of *L. monocytogenes* and the recurrent cross‐contamination of food products are largely attributed to the formation of biofilms on hard‐to‐clean harborage and the protection from biofilms against environmental stresses (Holch et al., 2013; Lunden, Autio, Markkula, Hellstrom, & Korkeala, 2003). However, the mechanisms underlying these processes are not clear enough to develop efficient strategies for biofilm prevention or disruption. Researches have begun uncovering the regulation of accessory gene regulator‐based quorum sensing (Agr QS) on virulent factors and the autoregulation at its own *agr* locus (Autret et al., 2003; Garmyn et al., 2012; Paspaliari, Mollerup, Kallipolitis, Ingmer, & Larsen, 2014; Riedel et al., 2009). This suggests that Agr QS orchestrates the pathogenesis and other stress adaptions of *L. monocytogenes* with multiple signal transduction pathways. In this study, our results reveal that genes for EPS synthesis, which is essential for biofilm formation, are tuned by Agr QS and a metabolic signal triggered by SAM. Critically, the results also show that the signals from Agr QS and SAM regulate the transcription of each other's components and that this link depends on the *L. monocytogenes* life modes (planktonic or sessile). The incorporation of clarified mechanisms by SAM signal and Agr QS in current views on the manipulation of *L. monocytogenes* biofilm development can be a good start point to improve the control strategies of this foodborne pathogen in food‐processing environments.

### 4.1 SAM signal enhances biofilm formation and upregulates *agr* gene transcription

In agreement with the effect of SRH (a SAM‐derived product in the AMC) on *L. monocytogenes* attachment (Challan Belval et al., 2006), we further confirm that a signal directly from SAM enhanced *L. monocytogenes* biofilm formation (Figure 2). These pieces of evidence support the metabolic role of AMC in the regulation of *L. monocytogenes* biofilm formation (Garmyn, Gal, Lemaitre, Hartmann, & Piveteau, 2009). Since SAM and its binding with riboswitches regulate the transcription of genes for the biosynthesis, transport, and utilization of amino acids, oligopeptides, and SAM itself (Loh et al., 2009; Winkler et al., 2003), it is conceivable that SAM signal controls nutrient availability and transduces metabolite‐binding events into genetic responses and thus precisely regulates cellular functions including biofilm formation. As our result showed SAM‐regulated expression of *agr* genes (Figure 3), we suggest that the regulation of SAM signal on biofilm formation is related to the transcription of *agr* genes.

Currently, autoregulation of the intrinsic regulator AgrA (Riedel et al., 2009; Rieu et al., 2007) and regulation of MouR, a GntR family of transcriptional factor (Pinheiro et al., 2018), are the two known regulatory mechanisms for the transcription of the *agr* locus. However, given our findings and the observation of reduced *agrD* expression in a mutant with a deletion of *sreA*, an RNA riboswitch SreA binding with SAM (Loh et al., 2009) is likely to be an alternative mechanism contributing to the transcription of *agr* locus. Although the *agr* locus is not the downstream mRNA of the seven putative SAM riboswitches in *L. monocytogenes* (Toledo‐Arana et al., 2009), it has been reported that SAM riboswitches could act as noncoding RNAs and regulate the expression of *trans*‐encoded target mRNAs. For example, SAM riboswitch SreA can decrease the gene expression and protein synthesis of the master virulence regulator PrfA by directly interacting with the 5' UTR of *prfA* gene (Loh et al., 2009). Further studies using RNA‐RNA gel shifts are needed to characterize the direct interaction between SAM‐binding SreA and individual genes in the *agr* locus. Nevertheless, indirect mechanisms may also contribute to the expression of *agr* genes in response to the SAM signal, such as via the decay of mRNA by ribonucleases (Baumgardt et al., 2017; Caron et al., 2012).

Intriguingly, our results (Figures 3 and 6a), together with previous findings (Autret et al., 2003; Rieu et al., 2007), reveal that the expression level of individual genes in the *agr* locus is unequal from one to another. It is an unusual observation for a cluster of genes under the control of a single promoter (Autret et al., 2003). A possible explanation could be discrepant mRNA stability of individual genes in *agr* locus (Rieu et al., 2007). It will be interesting to study whether this difference in mRNA stability of *agr* genes occurs on purpose for physiological functions or it is merely an artificial effect during experimental preparation. The experiments such as previously mentioned RNA‐RNA gel shifts to analyze the binding of SAM riboswitches to *agr* genes and a protein–DNA immunoprecipitation to identify the binding of ribonucleases to *agr* genes can help answer this question.

### 4.2 A regulatory network by the SAM signal and Agr QS for EPS synthesis

The classical biosynthesis of peptidoglycan is fundamental for the maintenance of biofilm structures (Freitas, Alves, & Reis, 2011; Rehm, 2010). Our qPCR results indicate that both the SAM signal and Agr QS have effects on peptidoglycan synthesis at the transcriptional level, but their targets are not the same (Figure 4). These data provide new insights into a precise regulation via nutrient availability and quorum sensing on EPS synthesis of *L. monocytogenes*. More specifically, we propose that *L. monocytogenes* perform a regulatory network based on the SAM signal and Agr QS to control different components in EPS synthesis pathway for overall biofilm development. However, future works, including the treatment of antibiotics or inhibitors for peptidoglycan synthesis and complement of target genes or signals in *agr* mutants, are required to directly link SAM‐ and Agr QS‐regulated EPS synthesis to biofilm formation.

### 4.3 Life mode‐dependent expression and regulation of Agr QS

Environmental niches and growth phases are crucial determinants of phenotypic heterogeneity in biofilms (van Gestel & Nowak, 2016). In line with the finding about the greater abundance of the QS peptide‐processing endopeptidase AgrB in attached sessile cells than in planktonic cells (Mata, Da Silva, Wilson, Lowe, & Bowman, 2015), we also found that the expression of *agrD* was greater in sessile cells compared to their planktonic counterparts (Figure 6). This suggests that the expression of Agr QS signal is life mode‐dependent. Regarding the transcriptional regulation via Agr QS, we found that Agr QS had a negative effect on the transcription of genes for SAM synthesis *(metK)* and methionine uptake (*lmo2417*) in planktonic life mode but on cysteine uptake (*lmo0135*) in sessile life mode (Figures 5b and 7). This suggests that Agr QS‐regulated functions are also life mode‐dependent.

The term quorum sensing emphasizes the concept that elevated concentrations of the QS signal enable a coordinated control of gene expression when the population reaches a quorum. That is, the primary function of QS system is to monitor an increase in the population density and to provide corresponding reactions (Platt & Fuqua, 2010). However, the dedication of Agr QS to population density sensing in the species of *L. monocytogenes* is controversial and Agr QS may contribute to non‐population‐dependent behavior (Garmyn et al., 2011; Riedel et al., 2009). Given the findings that SAM signal induced *agr* gene expression (Figure 2) and Agr QS inhibited the transcription of genes for SAM synthesis (Figures 5b and 7), it is possible that *L. monocytogenes* might utilize accumulation of Agr QS signal to respond to nutrient availability in the environment.

In addition to the effect of bacterial life modes, the greater alteration of *lmo0135* expression in Δ*agrD* relative to that in Δ*agrA* (Figure 5b) implies that the alteration of the signal precursor AgrD makes a greater effect on the transcription for cysteine than that of the regulator AgrA. The presence of this result would be unlikely if AgrA is the only downstream signal transducer for the AgrD signal. Thus, there might be two‐component systems other than AgrCA for the detection and transduction of the AgrD signal (Zetzmann, Sánchez‐Kopper, Waidmann, Blombach, & Riedel, 2016) or other intracellular regulators requiring AgrD as a cofactor for gene regulation.

### 4.4 A link between metabolism and biofilm formation

**Figure 8:**
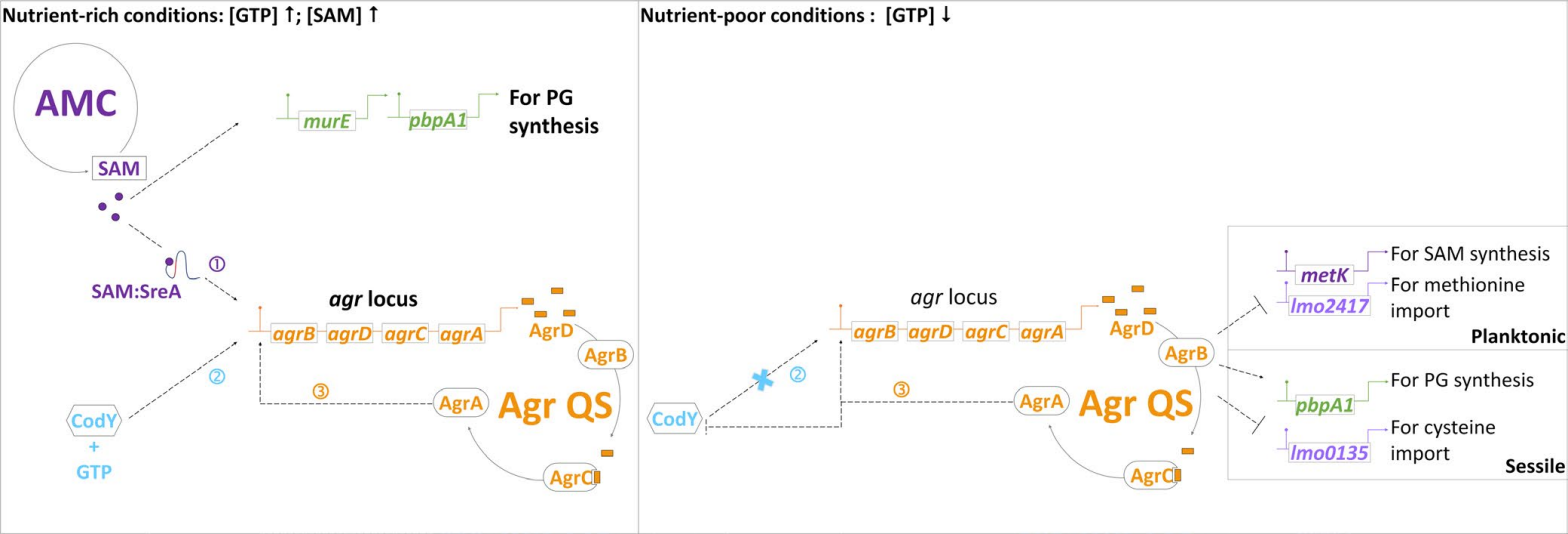
Agr‐CodY‐SAM regulatory network in *Listeria monocytogenes* under nutrient‐rich or nutrient‐poor conditions. SAM, CodY, and AgrA are three regulatory factors responsible for the transcription of the *agr* locus. (a) Under nutrient‐rich conditions with high concentrations of GTP and SAM, the expression of *agr* genes is upregulated by SAM with the RNA riboswitch SreA (1), CodY binding to GTP (2), and its autoregulation (3). Increased SAM also induces the transcription of *murE* and *pbpA1* for peptidoglycan synthesis. (b) Under nutrient‐poor conditions, the decrease in GTP concentration prevents CodY from being activated, which makes CodY no longer an activator for the *agr* locus. Depending on planktonic and sessile life modes of the bacteria, Agr QS influences the expression of different genes which are responsible for the synthesis of SAM signal and peptidoglycans. (SAM: *S*‐adenosylmethionine; AMC: activated methyl cycle; PG: peptidoglycan; Agr QS: accessory gene regulator‐based quorum sensing, a system including QS peptide precursor AgrD, QS peptide‐processing endopeptidase AgrB, kinase receptor AgrC, and response regulator AgrA)

Our findings together with those of prior reports provide evidence for the regulation of metabolite‐oriented Agr QS during biofilm development. The proposed mechanism includes a metabolic regulator CodY (Bennett et al., 2007; Elbakush, Miller, & Gomelsky, 2018; Garmyn et al., 2012, 2011) as well as SAM (this study) and its binding riboswitch SreA (Loh et al., 2009). These regulators could monitor the nutrient availability and mediate the expression of genes for EPS synthesis (Figure 8). We highlight that SAM signal and Agr QS interact with each other at the transcriptional level and they contribute to EPS synthesis through different routes.

Our data also show that Agr QS links to multiple metabolic genes and that these interconnections are activated in *L. monocytogenes* during a certain life mode. Since metabolic processes such as the metabolism of branched‐chain amino acids via CodY and sugar utilization in the phosphotransferase system have been reported to directly and indirectly interact with EPS synthesis and Agr QS (Bennett et al., 2007; Joseph et al., 2008; Lobel & Herskovits, 2016; Pinheiro et al., 2018), further investigation of the role of metabolic regulators such as CodY in Agr QS‐associated biofilm formation of *L. monocytogenes* is warranted.

As SAM and Agr QS are cooperative factors in the cross talk between *L. monocytogenes* methyl metabolism and EPS synthesis, it is suggested that the SAM synthase MetK, SAM‐dependent methyltransferases (Zhang & Zheng, 2016), and SAM‐mediated peptidoglycan synthesis are potential targets for antagonists (Yadav, Park, Chae, & Song, 2014) combined with Agr QS inhibitors (Fleming & Rumbaugh, 2017; Gray, Hall, & Gresham, 2013; Nakayama et al., 2009; Nguyen et al., 2012) to prevent or disrupt listerial biofilms in food‐processing environments.

## CONFLICT OF INTEREST

None declared.

## AUTHOR CONTRIBUTIONS

Ye‐Jia Lee curte the data, performed formal analysis involved in the investigation, contributed to methodology, wrote the original draft, reviewed and edited. Chinling Wang conceived the study, acquired the funding, involved in the investigation, contributed to methodology, performed project administration, provided resources, supervised the study, involved in validation and visualization process, and wrote, reviewed, and edited the manuscript.

## ETHICS STATEMENT

None required.

## Data Availability

All data generated or analyzed during this study are included in this published article.
